# Assessment of the knowledge and attitudes regarding HIV/AIDS among pre-clinical medical students in Israel

**DOI:** 10.1186/1756-0500-7-168

**Published:** 2014-03-20

**Authors:** Rotem Baytner-Zamir, Margalit Lorber, Doron Hermoni

**Affiliations:** 1 Faculty of Medicine, The Technion – Israel Institute of Technology, Haifa, Israel; 2 Autoimmune Disease Unit, Rambam Medical Center, Faculty of Medicine, The Technion – Israel Institute of Technology, Haifa, Israel; 3 Department of Family Medicine, Sharon-Shomron District, Clalit Health Services; Sackler Faculty of Medicine, Tel Aviv University, Ramat Aviv, Israel

**Keywords:** Knowledge, Attitudes, HIV, AIDS, Medical students

## Abstract

**Background:**

Today’s medical students are the future physicians of people living with HIV/AIDS (PLWHA). It is therefore essential that medical students possess the appropriate knowledge and attitudes regarding PLWHA. This study aims to evaluate knowledge and attitudes of pre-clinical Israeli medical students and to assess whether their knowledge and attitudes change throughout their pre-clinical studies.

**Methods:**

A cross-sectional study was conducted among all pre-clinical medical students from the four medical schools in Israel during the academic year of 2010/2011 (a total of 1,470 students). A self-administered questionnaire was distributed. The questionnaire sought student responses pertaining to knowledge of HIV transmission and non-transmission routes, basic knowledge of HIV/AIDS treatment and attitudes towards HIV/AIDS.

**Results:**

The study’s response rate was 62.24 percent. Knowledge among pre-clinical medical students was generally high and showed a statistically significant improvement as students progressed through their pre-clinical studies. However, there were some misconceptions, mostly regarding HIV transmission via breastfeeding and knowledge of HIV prevention after exposure to the virus. Students’ attitudes were found to include stigmatizing notions. Furthermore, the majority of medical students correlated HIV with shame and fear. In addition, students’ attitudes toward HIV testing and providing confidential medical information were contradictory to health laws, protocols and guidelines. Overall, no positive changes in students’ attitudes were observed during the pre-clinical years of medical school.

**Conclusion:**

The knowledge of pre-clinical medical students in Israel is generally high, although there are some knowledge inadequacies that require more emphasis in the curricula of the medical schools. Contrary to HIV-related knowledge, medical students’ attitudes are unaffected by their progression through medical school. Therefore, medical schools in Israel should modify their curricula to include teaching methods aimed at improving HIV-related attitudes and adherence to medical professionalism.

## Background

HIV/AIDS has been a global pandemic for the last 30 years, and its spread has yet to be contained. However, due to the availability of antiretroviral therapy, today HIV is becoming a chronic disease, which means that more physicians from every medical field will encounter HIV-infected individuals throughout their medical careers. This obliges all medical staff to have both sufficient and correct knowledge regarding HIV/AIDS and a professional attitude towards the disease and the patients, unaffected by fears, stigma and misconceptions. The spread of HIV in any community is in part determined by its members’ knowledge concerning safe sexual practices and prevention of HIV transmission. It is highly important that medical students be knowledgeable about HIV/AIDS and have positive attitudes towards PLWHA, so that they can become better physicians who will conduct themselves according to the highest standards of both medical knowledge and medical professionalism. A better understanding of the students’ prior knowledge and attitudes towards HIV/AIDS will serve as a tool to create better educational programs dealing with stigma and encouraging empathy towards patients.

HIV content is taught in Israeli medical schools and includes similar content in all four schools. However, the medical schools curricula focuses mainly on matters such as the pathogenesis of HIV infection and the affects it has on its host, in courses like virology, cell biology, immunology etc. In all four medical schools, there are only 3–4 hours each pre-clinical year dedicated to subjects such as ethics and medical legal issues. To our knowledge there are no specific programs in any of the four schools dedicated to addressing and dealing with stigma toward specific patient populations.

Few studies on knowledge and attitudes regarding HIV/AIDS have been reported in Israel, and none of them have addressed the question of basic HIV/AIDS knowledge or attitudes towards HIV/AIDS among Israeli medical students. Therefore, the aim of this study was to evaluate the knowledge and attitudes of pre-clinical medical students in Israel towards HIV/AIDS, and to determine whether there is a change in knowledge or attitudes throughout the three pre-clinical years of medical school.

### Research questions

This study will attempt to answer the following questions: 1) What is the HIV/AIDS knowledge among pre-clinical medical students? 2) What are the current attitudes regarding PLWHA among pre-clinical medical students in Israel? 3) Is there a change in knowledge and attitudes throughout the three pre-clinical years of medical school?

## Methods

### Study design and setting

The study conducted was a questionnaire-based, cross-sectional study designed to assess knowledge and attitudes regarding HIV/AIDS among pre-clinical medical students in Israel. The study was carried out between November 2010 and January 2011 at all four medical schools that existed in Israel at the time (there are now five medical schools in Israel).

Medicine in Israel is taught over six academic years––the first three years are pre-clinical, while years four to six are clinical––followed by one year of internship. One of the medical schools at the time the study was conducted also had a four-year program, followed by one year of internship, which is intended for students with BA degrees prior to their enrolment in medical school. In the four-year program, the first two years are the pre-clinical years, which are equivalent to the first, second and third year of the six-year program. For the purpose of this study, the first and second years of the four-year program were considered equivalent to the first and second years of the regular six-year program, respectively. The curriculum is similar in the four-year program and the six-year program, and the content regarding HIV/AIDS is similar as well.

### Sample

The study cohort included all medical students in Israel in their pre-clinical years. In the academic year of 2010/2011, there were 1,470 medical students in their pre-clinical years studying in Israel. Nine hundred and fifteen students answered the questionnaire, a total response rate of 62.24%.

### Instrument

The study instrument was a self-administered questionnaire that was constructed of questions derived from several previously validated questionnaires that were administered as part of relevant previous studies [1-5]. In order to assess the content validity of the questionnaire, it was assessed by four medical doctors who specialised in the field of infectious diseases, with a specialty in HIV/AIDs. After the content validation of the questionnaire, and performing the necessary changes, a pre-test of the questionnaire was performed in order to assess the questionnaire’s reliability. The pre-test was performed one school year prior to the execution of the study, and included 41 third-year medical students attending The Technion – Israel Institute of Technology’s medical school, who would not be considered part of the study group at the time the study would be conducted. The internal validity (alpha Cronbach) of the questionnaire was 0.875.

The validated questionnaire consisted of four parts: socio-demographic information; background of previous exposure to information regarding HIV/AIDS and to HIV-positive individuals; knowledge and attitudes (see Additional file 1 for the complete questionnaire).

HIV/AIDS knowledge was measured with a 17-item set of questions covering knowledge of transmission and non-transmission routes of HIV and basic knowledge of HIV/AIDS treatment (treatment for prevention of HIV infection after exposure and for prolonging life expectancy in HIV-positive individuals). Participants could reply using one of three possible responses to each statement in the knowledge portion: ‘yes’ , ‘no’ or ‘don’t know’. The attitudes portion of the questionnaire consisted of 33 items that were measured on a four-point Likert-type scale ranging from 1 (strongly agree) to 4 (strongly disagree). The 33 items regarding attitudes were divided into five categories: 1) desire for knowledge regarding HIV/AIDS; 2) general stigma towards HIV/AIDS; 3) medicine and stigma towards HIV/AIDS; 4) emotions and fears regarding HIV/AIDS; and 5) HIV/AIDS-related medical protocol.

The questions were translated from English to Hebrew and translated back into English by a certified translator. Modifications were made to adjust the questionnaire to the study population (i.e., pre-clinical medical students in Israel).

### Statistical analysis

All the data from the questionnaires was entered into Microsoft Excel® and later downloaded to SPSS® for Windows release 19 (SPSS Inc., Chicago, IL, USA) for statistical analysis.

To compare continuous variables either t-tests or ANOVA were used, while Pearson’s Chi-square was used to analyse categorical variables. A p-value < 0.05 was considered as statistically significant.

### Ethics

Permission to conduct the study was obtained from The Technion – Israel Institute of Technology, the Bruce and Ruth Rappaport Faculty of Medicine MD Thesis Committee (each medical school has its own committee, which follows guidelines created by the Israeli Ministry of Health). The MD Thesis Committee approved the research proposal and ethics of the study.

The questionnaires were distributed by the same researcher in all four medical schools following a short explanation regarding the purpose of the study. The questionnaires were anonymous, and participation in the study was completely voluntary, with students having the option of declining to answer the questionnaire. Students who decided to answer the questionnaire were considered as having given their consent to participate in the study.

## Results

The mean age of the study population was 23.6 years (range 17–36). The demographics of the four medical schools according to gender, ethnicity and year are presented in Table 1.

**Table 1:** Demographics of schools according to gender, ethnicity and year

**Variable**	**The Technion – Israel Institute of Technology (n=200)**	**Tel Aviv University (n=289)**	**The Hebrew University of Jerusalem (n=276)**	**Ben-Gurion University of the Negev (n=150)**	**Total (n=915)**
Gender:					
% Male	44.5	36	47.1	49.3	43.4
% Female	54.5	63.7	52.9	50.7	56.3
% NA	1	0.3	0	0	0.3
Ethnicity:					
% Jewish	67.5	86.2	89.9	91.3	84
% Non-Jewish	32	13.8	9.8	8.7	15.8
% NA	0.5	0	0.3	0	0.2
Year:					
% First year	36.5	40.83	45.29	36.66	40.5
% Second year	37	36.33	31.88	28.67	33.9
% Third year	26.5	22.84	22.83	34.67	25.6

The percentage is calculated from the total number of medical students in years1–3 at each university.

NA represents students who did not answer the specific question.

Non-Jewish includes all the students who declared themselves to be Muslim, Christian, Druze, Bedouin or Other (8%, 5.2%, 1.5%, 0.4%, and 0.5% respectively).

### Knowledge

#### Sources of HIV/AIDS information

Participants were allowed to select more than one option. The most common sources were newspapers (80.3%), TV/radio (77.7%) and publications such as ads and flyers on the subject (63.5%). Medical literature was a less popular source (40.9%), along with teachers (38.3%), doctors (38.1%), books and magazines (32.3% and 30.5% respectively). The least common sources of HIV/AIDS information were friends and parents (29.7% and 20.5% respectively); 9.1% of the responders reported other forms of HIV/AIDS information, mostly noting the Internet.

#### Exposure to information about HIV/AIDS and PLWHA

Exposure to information about HIV/AIDS during medical school statistically increased from year to year: 15.8%, 75.7% and 93.1%, from first, second to third year respectively (n = 906, p < 0.001). Only 5.1% of students stated they had met or spoken with PLWHA during medical school.

#### The most common cause of HIV transmission in Israel

Intravenous drug users (IDUs) were selected as the most common cause of HIV transmission in Israel by the students (40.9%). The second most common cause was heterosexual relations (25.5%) and the third was men who have sex with men (MSM) (19.1%).

**Figure 1:**
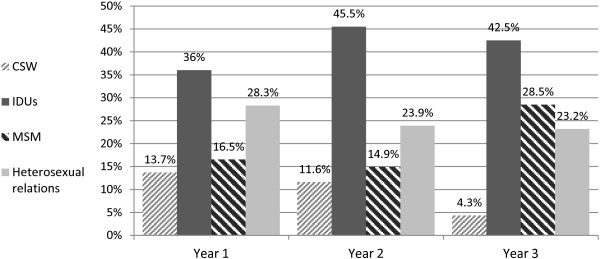
**The most common cause for contracting HIV in Israel by medical year.** The students could only pick one of several options, including commercial sex (CSW), intravenous drug users (IDUs), men who have sex with men (MSM), heterosexual relations, tattoos and piercings, dental work and mother to foetus transmission. Shown in this table are the four most common answers selected by the study population (n = 797). CSW: n = 84, p = 0.002; IDUs: n = 326, p = 0.349; MSM: n = 152, p < 0.001; heterosexual relations: n = 203.

Figure 1 demonstrates the most common cause for contracting HIV in Israel by medical year. When comparing the three medical years, a statistically significant decline (p = 0.002) was found from first, second, to third year in the percentage of students who thought CSW was the most common cause of HIV infection in Israel (13.7%, 11.6% and 4.3% respectively). Findings showed that 16.5% of first-, 14.9% of second- and 28.5% of third-year students thought MSM was the most common cause of HIV in Israel (p < 0.001). IDU was chosen as the most common cause of HIV in Israel by all three medical years: 36% of first-year, 45.5% of second-year and 42.5% of third-year students (p = 0.349). There was a non-statistically significant decline (p = 0.325) from first to third year in the percentage of students who regarded heterosexual relations (28.3%, 23.9% and 23.2% respectively) as the most common cause for contracting HIV in Israel.

#### HIV/AIDS knowledge of transmission, non-transmission and basic treatment

Table 2 shows the percentage of students who correctly answered knowledge questions regarding routes of HIV/AIDS transmission, non-transmission and basic knowledge of HIV/AIDS treatment. Knowledge of HIV transmission and non-transmission routes was generally high; more than 80% of students knew the correct answer to most questions. However, only 36.6% of students knew HIV could be transmitted from mother to child via breast feeding. Knowledge of the non-transmission routes via saliva and mosquito bites was lower than expected, 75.2% and 72.8%, respectively. Students’ basic knowledge of HIV treatment showed substantial misconceptions with respect to the possibility of preventing HIV infection after exposure. More than 40% of students did not know that HIV infection can be prevented after unprotected sexual intercourse or after a prick from an infected needle. More than 50% of students did not know that prevention of HIV transmission from mother to child during pregnancy and labour was possible.

**Table 2:** Frequency and percentages of knowledge regarding routes of HIV/AIDS transmission, non-transmission and HIV treatment

**Knowledge items**	**% of students who answered correctly**
*Routes of transmission*	
Sexual contact (n = 915)	100
Contaminated needles & syringes (n = 914)	99
Blood transfusion (n = 911)	94.2
From mother to child during pregnancy & labor (n = 905)	91.9
Tattoos and piercings (n = 906)	81.6
From mother to child during breastfeeding (n = 908)	36.6
*Routes of non-transmission*	
Hand shaking (n = 908)	99.3
Hugging (n = 906)	99.1
Sharing utensils with someone who has HIV/AIDS (n = 907)	89.6
Using the same toilet as someone who has HIV/AIDS (n = 908)	88.9
Saliva from someone who has HIV/AIDS (n = 892)	75.2
Mosquito bites (n = 904)	72.8
*Basic knowledge of HIV/AIDS treatment*	
HIV treatment prolongs the life expectancy of HIV-positive patients (n = 908)	93.5
HIV treatment decreases the chances of infection after unprotected sexual intercourse (n = 904)	57.9
HIV treatment decreases the chances of infection after a prick from an infected needle (n = 903)	53.6
HIV transmission from mother to child during pregnancy & labour can be prevented (n = 907)	46.2

The percentage represents the number of students who answered correctly out of all the students who answered the particular question (n). Students who did not answer a question were considered absent from the statistic regarding that particular question. The selection of ‘don’t know’ as an answer was considered as a wrong answer.

The percentage represents the number of students who answered correctly out of all the students who answered the particular question (n). Students who did not answer a question were considered absent from the statistic regarding that particular question. The selection of ‘don’t know’ as an answer was considered as a wrong answer.

Table 3 shows the percentages of correct responses to knowledge items by medical school year. When comparing between the medical years, knowledge of the non-transmission route by mosquito bites statistically improved (p < 0.001) from first, second to third year (69.8%, 72.6% and 77.7%, respectively). A statistically significant improvement was found in all four items of basic knowledge of HIV/AIDS treatment. However, knowledge of HIV infection prevention was unsatisfactory, ranging from 58% to 70% among third-year medical students.

**Table 3:** Percentages of correct responses to knowledge items by medical school year

**Knowledge items**	**First year %**	**Second year %**	**Third year %**	**P value**
*Routes of transmission*				
Sexual contact (n = 915)	100	100	100	–
Contaminated needles & syringes (n = 914)	98.9	98.7	99.6	0.397
Blood transfusion (n = 911)	94.6	91.6	97	0.54
From mother to child during pregnancy & labour (n = 905)	91	91.5	94	0.577
Tattoos and piercings (n = 906)	80.4	82	82.8	0.781
From mother to child during breastfeeding (n = 908)	36.3	33.8	40.6	0.498
*Routes of non-transmission*				
Hand shaking (n = 908)	99.2	99.4	99.6	0.5
Hugging (n = 906)	99.2	99	99.1	0.978
Sharing utensils with someone who has HIV/AIDS (n = 907)	87.7	90.9	91	0.219
Using the same toilet as someone who has HIV/AIDS (n = 908)	86.9	89.6	91	0.489
Saliva from someone who has HIV/AIDS (n = 892)	73.6	74.8	78.3	0.681
Mosquito bites (n = 904)	69.8	72.6	77.7	<0.001
*Basic knowledge of HIV/AIDS treatment*				
HIV treatment prolongs the life expectancy of HIV-positive patients (n = 908)	89.4	94.1	99.1	<0.001
HIV treatment decreases the chances of infection after unprotected sexual intercourse (n = 904)	50	57.7	70.4	<0.001
HIV treatment decreases the chances of infection after a prick from an infected needle (n = 903)	44.3	53.4	68.5	<0.001
HIV transmission from mother to child during pregnancy & labour can be prevented (n = 907)	38.6	46.4	57.9	<0.001

The percentage represents the number of students who answered correctly out of all the students who answered the particular question (n). Students who did not answer a question were considered absent from the statistic regarding that particular question. The selection of ‘don’t know’ as an answer was considered as a wrong answer.

### Attitudes

#### Desire for knowledge regarding HIV/AIDS

Most of the participants (94.8%) stated that they would like to know more about HIV/AIDS. In addition, the majority of students (59.1%) felt they did not acquire enough information from their professional education in order to work safely with PLWHA.

When comparing among the three medical years, no significant differences were found regarding students’ desire for knowledge or their feelings of preparedness for safe professional contact with PLWHA.

#### General stigma regarding HIV/AIDS

**Figure 2:**
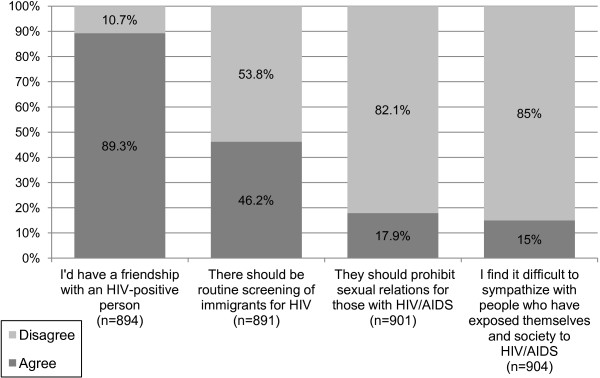
**General stigma regarding HIV/AIDS.** The percentage represents the number of students who agreed (dark grey), or disagreed (light grey) out of all the students who answered the particular question (n). Students who did not answer a question were considered absent from the statistic regarding that particular question. Students who stated they ‘strongly agree’ or ‘agree’ with a statement are shown as ‘agree’ (dark grey), and students who stated they ‘strongly disagree’ or ‘disagree’ with a statement are shown as ‘disagree’ (light grey).

The students’ answers to several of the questions regarding general HIV/AIDS stigma are presented in Figure 2. Almost all the participants in the study disagreed with blatant stigmatizing statements such as ‘only MSM can contract HIV’ (98.8%), ‘people who have HIV/AIDS get what they deserve’ (98.7%), and ‘people with AIDS should be quarantined’ (98%). A positive finding was that nearly 90% of students stated they would have a friendship with an HIV-infected person. About 46% of students thought there should be routine screening of immigrants for HIV, while 17.9% of students felt that sexual relations should be prohibited for those with HIV/AIDS. Results showed that 15% of participants found it difficult to sympathise with PLWHA, whom they viewed as having exposed themselves and society to HIV/AIDS.

When comparing among the three medical years, all items in Figure 2 except for one had no statistically significant differences. Study findings demonstrated a statistically significant decline (p = 0.001) from first (22.7%), second (17.4%) to third year (10.8%) in the percentage of the students who stated that ‘sexual relations should be prohibited for those with HIV/AIDS’.

#### Medicine and stigma regarding HIV/AIDS

**Figure 3:**
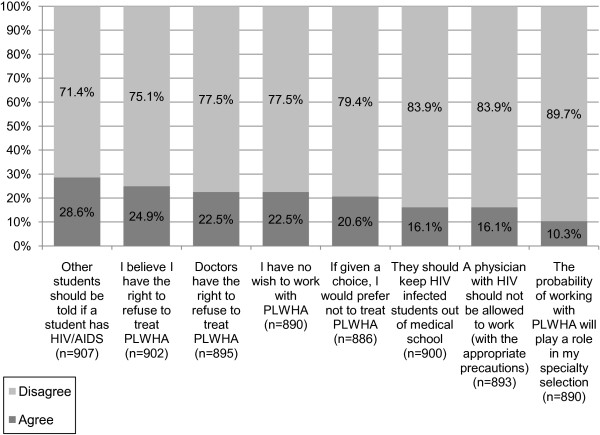
**Medicine and stigma regarding HIV/AIDS.** The percentage represents the number of students who agreed (dark grey), or disagreed (light grey) out of all the students who answered the particular question (n). Students who did not answer a question were considered absent from the statistic regarding that particular question. Students who stated they ‘strongly agree’ or ‘agree’ with a statement are shown as ‘agree’ (dark grey), and students who stated they ‘strongly disagree’ or ‘disagree’ with a statement are shown as ‘disagree’ (light grey).

The results of the attitudes items regarding medicine and stigma towards HIV/AIDS are presented in Figure 3. Findings show the presence of stigmatizing attitudes towards PLWHA among medical students, which range from a tenth up to approximately a third of participants. Nearly 30% of participants felt that other students should be notified if one of the students was HIV positive. Nearly a quarter of students stated they believed they had the right to refuse to treat PLWHA, however, only 4.1% stated that they would actually refuse to treat PLWHA. More than a fifth of the students believed that doctors had the right to refuse to treat PLWHA. In addition, more than a fifth of participants stated they had no wish to work with PLWHA. More than 20% of participants stated they would prefer not to care for PLWHA if given the choice. More than 16% thought HIV-positive students should be kept out of medical school, and that an HIV-positive physician should not be allowed to work, even with the appropriate precautions. More than 10% of students stated that the possibility of working with PLWHA would play a role in their choosing a medical specialty.

When comparing among students from the three medical years, only one of the items in Figure 3 showed a statistically significant change. A statistically significant difference was found regarding the statement that ‘if given a choice, I would prefer not to treat PLWHA’: 20.4%, 16.8% and 26% of first-, second- and third-year students respectively agreed with this statement (p = 0.036).

#### Fears and emotions regarding HIV/AIDS

**Figure 4:**
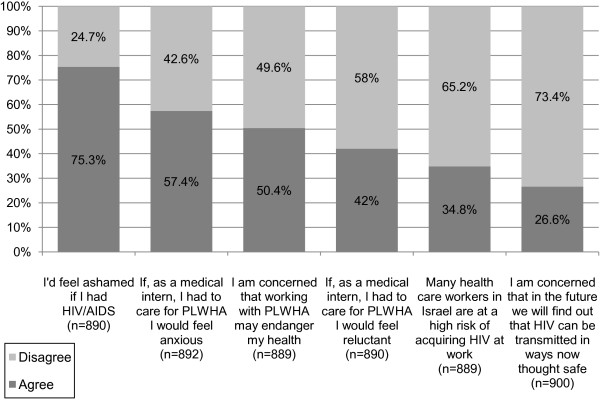
**Fears and emotions regarding HIV/AIDS.** The percentage represents the number of students who agreed (dark grey), or disagreed (light grey) out of all the students who answered the particular question (n). Students who did not answer a question were considered absent from the statistic regarding that particular question. Students who stated they ‘strongly agree’ or ‘agree’ with a statement are shown as ‘agree’ (dark grey), and students whom stated they ‘strongly disagree’ or ‘disagree’ with a statement are shown as ‘disagree’ (light grey).

Participants’ answers to items regarding fears and emotions toward HIV/AIDS are presented in Figure 4. Findings show that participants strongly view HIV/AIDS as a condition to be ashamed of, with the majority of students (75.3%) stating that they would feel ashamed if they were HIV positive. In addition, the majority of students (57.4%) stated they would feel anxious if, as interns, they had to care for PLWHA. About 50% of students were concerned that working with PLWHA might endanger their health. More than 40% of students stated that as interns they would feel reluctant to treat PLWHA. More than a third of participants believed that many of the health-care workers in Israel were at high risk of acquiring HIV at work; 26.6% were concerned that in the future it would be found that HIV infection could be transmitted in ways now thought to be safe.

When comparing the three medical years, no statistically significant changes were found in students attitudes concerning fears and emotions towards HIV/AIDS.

#### HIV/AIDS-related medical protocol

**Figure 5:**
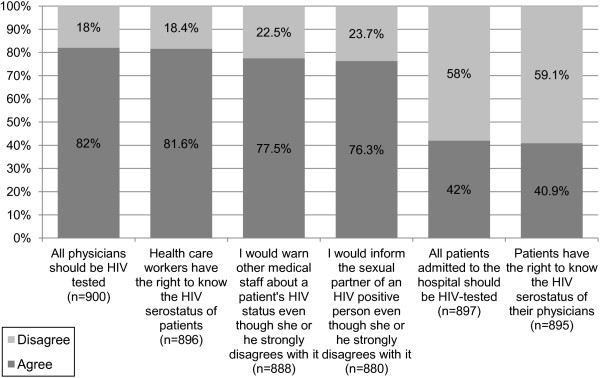
**HIV/AIDS-related medical protocol.** The percentage represents the number of students who agreed (dark grey), or disagreed (light grey) out of all the students who answered the particular question (n). Students who did not answer a question were considered absent from the statistic regarding that particular question. Students who stated they ‘strongly agree’ or ‘agree’ with a statement are shown as ‘agree’ (dark grey), and students who stated they ‘strongly disagree’ or ‘disagree’ with a statement are shown as ‘disagree’ (light grey).

Results of the attitudes section concerning HIV/AIDS-related medical protocol are presented in Figure 5. More than 80% of medical students thought all physicians should be HIV tested and that health-care workers had the right to know their patients’ HIV status. Close to 80% stated they would warn other medical staff about a patient’s HIV status against that patient’s wishes and that they would inform the sexual partner of an HIV-positive person against the patient’s wishes. More than 40% agreed that all patients admitted to the hospital should be tested for HIV and more than 40% believed patients had the right to know the HIV status of their physicians.

When comparing among medical years, findings showed statistically significant differences regarding two of the items presented in Figure 5. A statistically significant increase was found from first, second to third year, 78.7%, 80.9% and 87% respectively (p = 0.039), in the percentage of students who thought health-care workers had the right to know the HIV serostatus of patients. A statistically significant increase was demonstrated in the percentage of students who stated they would warn other medical staff about a patient’s HIV status against that patient’s wishes (74.4%, 75.8% to 84.6% respectively, p = 0.011).

## Discussion

The purpose of this study was to assess the knowledge and attitudes of pre-clinical medical students towards HIV/AIDS. To our knowledge, this study is novel as no previous study has been performed in Israel that includes a nationwide analysis of pre-clinical medical students’ knowledge and attitudes toward HIV/AIDS.

When observing the different sources of HIV information selected by the students, the popular media (such as newspapers, TV and radio) was the most popular choice. This data is in accordance with data shown in previous studies conducted both in Israel and other countries [2,6-10]. Several of these studies encompass the general population and not representatives of the medical professions, including in the study by Feldman and colleagues [7] of a national sample of young Israeli men and women. One can conclude that pre-clinical medical students gain HIV/AIDS information from the same sources as the general public in Israel. This finding is not surprising since the popular media has always played a prominent role in creating awareness of various health matters, including HIV.

Many of the students who cited other sources of information reported the Internet as one. It is reasonable to assume that if ‘the Internet’ had been given as an option for a possible source of HIV/AIDS information, it would have been a popular source of information among the medical students given its popularity and availability to the public.

Students’ exposure to HIV/AIDS information increased from first year to third year. This correlates with the notion that as students progress through medical school they are exposed to more medical information, including information on the subject of HIV/AIDS.

The incidence of new HIV/AIDS infections in Israel in 2011 was 58 new cases per million, a lower incidence than in most western European and North American countries [11]. HIV in Israel is influenced mainly by immigration from countries with generalised HIV epidemics such as African countries and the former USSR [12]. During the period between 1981 and 2011, there was an increase in the number of new HIV/AIDS reported cases via MSM transmission, making it the most common cause for contracting HIV/AIDS in Israel (not including PLWHA originating from countries with a generalised HIV epidemic) [11].

The majority of medical students who participated in this study selected IDU as the most common cause of HIV/AIDS transmission in Israel, followed by heterosexual relations and MSM. The view of IDUs as the leading cause of HIV/AIDS in Israel was unchanged when comparing among the three medical years. These findings are not surprising when considering the well-known stigma towards IDUs and the linkage between IDU and HIV/AIDS, as demonstrated by various studies [13-20]. Sayles and colleagues [19] used focus groups to explore experiences and perceptions of stigma in a diverse group of women and men living with HIV. Findings showed that PLWHA are seen as ‘contaminated’ and linked to the stereotypes of promiscuity, drug use and homosexuality.

In a study among Thai nursing students performed by Chan and colleagues [20], IDU was found to be the most stigmatizing co-characteristic of HIV/AIDS. They hypothesised that the moral failings represented by HIV/AIDS could be derived from the moral failings represented by IDU. An intrinsic association between IDU and HIV as cause and effect in the minds of medical students might explain our findings. In addition, one might also conclude from our findings that there is a lack of knowledge among medical students regarding the epidemiology of HIV infection in Israel.

The overall knowledge of HIV transmission and non-transmission routes was high among students. However, there were several misconceptions, the most prominent being with regard to transmission via breastfeeding, with only 36.6% knowing that breastfeeding was one of the routes of HIV transmission. Furthermore, this knowledge had not improved throughout the three pre-clinical years. Knowledge of breastfeeding as a transmission route for HIV was even lower in a study among first-year nursing students in Turkey, 12.5%, with knowledge improving to 43.2% after an HIV education program [9]. In a study among Korean dentists, only 28% knew breast milk is a transmission route for HIV [21]. Knowledge was less than expected from medical students regarding the non-transmission routes via saliva and mosquito bites, 75.2% and 72.8% respectively. In a study among first year medical students in the city of Madras in India, 86.8% of students knew HIV cannot spread by mosquitoes [2]. Similar results were also demonstrated in a study by Najem and Okuzu [22] among first- and second-year medical students and in a study among fourth-year medical students at Zagreb University. In a study by Chemtob and colleagues among Israeli adults, 34% of participants thought mosquitoes could spread HIV, and 29% thought HIV can be spread via saliva [23].

There were considerable lacunae in the students’ basic knowledge of HIV treatment and prevention methods in all but one of the four items, with almost all participants knowing HIV treatment can prolong the life expectancy of PLWHA. However, knowledge regarding prevention of HIV infection was insufficient. Although findings demonstrated a statistically significant improvement in knowledge of HIV prevention, one would expect knowledge of third-year students to reach close to 100%, a fact not demonstrated in this study. This is concerning since fourth-year medical students in Israel start their clinical rotations during which they are exposed to patients.

One can assume that the fact that overall there is improvement in knowledge throughout the pre-clinical years may also imply that the improvement in knowledge continues during the clinical years. Turhan et al. compared the HIV knowledge of first-year students to final-year students from faculties of medicine, dentistry and medical technology vocational training school [24]. Final-year students were indeed found to have higher levels of knowledge when compared to first-year students. In a study by Chew and Cheong [25] at a public university in Malaysia, knowledge scores were significantly higher for clinical compared to pre-clinical medical students.

However, when medical students come into contact with patients, they must already be fully aware of the universal precautions, of proper prevention methods such as post-exposure prophylaxis, and of hospital safety protocols. This important information should be provided to medical students early in their pre-clinical studies and repeated later in order to consolidate the data in the students’ minds. Thus, all students beginning their first clinical year will be well versed in these areas. In addition, misperceptions may result in an adverse impact on medical students’ willingness to be in close contact with PLWHA, thus interfering with the quality of medical care these patients receive. Najem and Okuzu concluded that misperceptions may result in an adverse impact on the willingness of students to have close contact with PLWHA, which in turn may interfere with high-quality medical care for PLWHA [22].

Findings revealed a universal desire among students to gain further HIV/AIDS knowledge, unchanged through the pre-clinical years. In addition, most of the participants felt their current level of professional education was not sufficient to work safely with PLWHA, a feeling unchanged during the pre-clinical years. In a study among final-year medical and pharmacy students, Ahmed and colleagues found most students showed fear of incompetence in the treatment, care or even counselling of patients [26]. Students had doubts about the level of competence of their educational training in safely dealing with PLWHA. These findings are similar to those found in studies among medical students in other countries [2-4,27].

Findings demonstrated that the majority of students have positive attitudes towards PLWHA. However, the presence of stigmatizing attitudes towards PLWHA that were demonstrated in this study cannot be ignored. Close to half of the students believed there should be routine screening of immigrants for HIV. Nearly 18% agreed that PLWHA should be prohibited from having sexual relations. Furthermore, almost a quarter of students believed they had a right to refuse to treat PLWHA. Similarly, a study among second-year medical students found one-third of students believed they had this right [3]. More than a fifth of Israeli students thought doctors had the right to refuse to treat PLWHA, the same outcome as in studies among medical students in England and Croatia [4,5].

These results are alarming, since they show an undeniable presence of prejudice among the medical student population. Nearly all stigmatizing attitudes in this study remained unchanged and persisted throughout the pre-clinical years. Bernstein and colleagues surveyed second- and third-year medical and dental students before and after they completed a year of required clinical training [28]. Findings showed that a substantial minority of students did not acknowledge a responsibility to treat all patients, regardless of their HIV status. Furthermore, these attitudes persisted over time and were hardly influenced by the students’ clinical exposure.

The majority of medical students made a strong link between HIV and shame. This concept of HIV as something to be ashamed of can be attributed to the existence of stigmatizing notions towards HIV––the association of HIV with particular groups such as IDUs and CSW, to immoral or promiscuous behaviour [13,15,17,20], and to a fear of the social rejection that might follow a positive HIV diagnosis. For example, a study among medical students in China concluded that the stigma of IDU and CSW was embedded within being HIV-positive [18]. In a study among HIV-positive adults, individuals were seen as ‘contaminated’ and linked to the stereotypes of promiscuity, drug use and homosexuality [19].

Study findings showed that medical students have high levels of fear towards HIV/AIDS. Nearly 35% of students felt their future occupation as health-care workers was placing them at a high risk of occupational HIV infection. This perception of risk is highly exaggerated as actual risk is quite small when implementing the proper precautions. In actuality, the risk to health-care workers of occupationally acquiring HIV infection after percutaneous exposure to HIV-infected blood is approximately 0.36% [29,30]. Nevertheless, concerns and fears regarding the risk of acquiring HIV infection occupationally among medical students, physicians and other health-care professionals are well documented in the medical literature [3,9,10,26-28,31-33].

A study among second-year medical students revealed that more than 60% expressed concern that working with PLWHA might be hazardous [3]. Another study among final-year medical students found that more than 70% showed reservations that working with PLWHA might endanger their health [26]. Fear of occupational HIV transmission was also prevalent in a study among health-care providers––nearly 40% were afraid of acquiring HIV during the course of their work [32].

There was no change in the levels of fears or emotions regarding HIV/AIDS during the three medical years. It can be concluded that feelings of fear and the accompanying emotions towards HIV/AIDS are not influenced by the improvement in HIV/AIDS-related knowledge. Previous studies have shown the existence of stigmatizing notions and the persistence of fear towards HIV/AIDS among medical students in their pre-clinical and clinical years as well, and among those currently working in the medical professions, i.e., doctors, nurses and dentists [22,24,28,33-38]. Evidence of stigmatization against a patient population by those in the medical profession cannot be ignored, for such attitudes affect the way patients are treated, and can impact the quality of care they receive.

A concerning finding was that students showed a nearly complete lack of awareness regarding issues of HIV protocol in Israel. The majority of students thought all physicians should be tested for HIV. Similar results were shown in a study by Mohsin, Nayak and Mandaviya among first- and second-year medical students [27] and in a study by Ahmed, Hassali, Bukhari and Sulaiman among final-year medical and pharmacy students [26]. In actuality, the Israeli Health Department’s protocols regarding health-care workers and HIV states that there is no justification for performing HIV screening for all health-care workers, and that health-care workers cannot be obligated to be HIV tested [39].

Findings demonstrated that more than 40% of students felt all patients admitted to the hospital should be tested for HIV with similar trends demonstrated in studies among medical students, pharmacology students and nurses [3,25,26,40]. In one study, approximately 90% of medical students stated that all patients admitted to the hospital should be HIV tested [27]. In reality, there is no medical necessity to perform routine HIV screening for all patients admitted to hospital for it does not reduce the risk of occupational exposure. Furthermore, routine HIV testing is not a valid economic alternative to universal precautions [41].

More than three quarters of medical students believed they had the right to inform the sexual partner of an HIV-positive patient against that patient’s wishes. An even larger percentage of students thought they had a right to disclose this information to other medical staff. Mohsin and colleagues found that 77% of first- and second-year medical students would inform the spouse of PLWHA, even if forbidden to do so by the patient [27]. In another study, more than 50% of medical students stated they would warn other medical staff about a patient with HIV, even if the patient disagreed [5]. This belief is contradictory to Israeli law regarding medical confidentiality (The Patients’ Rights Law, 1996), which states that physicians and other health-care workers have to keep all patient information confidential. They can only give this information to another party if the patient consents or if an ethics committee approves. The same law states that health-care workers can provide confidential medical information to medical colleagues only when these colleagues are scheduled to render medical treatment to the patient.

Knowledge regarding patient confidentiality and HIV/AIDS health protocols in Israel is highly important for all those who work in the medical system. It is crucial that this information be taught and discussed at length throughout medical school so that students, whether starting their clinical studies or beginning their work as interns and residents, are well prepared, and know their rights and their duties.

### Study limitations

One known limitation of a self-administered questionnaire is that respondents may provide the answers they believe to be the most suitable and desirable by the researchers, and not necessarily coming from their own conviction. During the distribution of the questionnaires to participants, the researcher attempted to minimise this limitation by declining to answer questions or respond to enquiries regarding the different questionnaire items.

## Conclusion

Pre-clinical medical students in Israel today still have gaps in their knowledge regarding the subject of HIV/AIDS, especially when concerning transmission via breast milk and the possibility of preventing HIV infection after exposure to the virus. Although the results of this study demonstrated an overall improvement in knowledge among students as they progressed through their pre-clinical studies, the reality is that HIV/AIDS knowledge is not sufficiently comprehensive among students prior to starting their clinical years. This study proves the existence of stigmatizing notions and fear among medical students towards PLWHA. Furthermore, the majority of medical students associated HIV with shame and expressed views and attitudes that were contrary to medical protocol and confidentiality.

The most alarming finding was that, unlike the improvement in knowledge throughout medical school, there was little change in the medical students’ attitudes towards HIV/AIDS. This finding emphasises the discrepancy between knowledge of HIV/AIDS and attitudes toward HIV/AIDS, and should focus the attention of the medical schools in Israel so they can act to improve future doctor-patient relationships. Doctors today need to possess sufficient knowledge of HIV/AIDS and have a non-judgmental attitude toward PLWHA so that these patients receive the optimal medical care they deserve without the influence of fear and stigma.

The results of this study show the importance and necessity of medical courses that deal with the subject of HIV/AIDS, focusing not just on furthering HIV knowledge but also on dealing with stigma, anxiety and misperceptions about HIV/AIDS and PLWHA. In addition, it is extremely important to conduct these courses before the students start their clinical studies so that correct and sufficient knowledge and attitudes, which are required when dealing with patients, are already well embedded in the students’ minds by that time.

## Competing interests

The authors declare that they have no competing interests.

## Authors’ contributions

RBZ conceived of the study, participated in its design and coordination, conducted the acquisition, input and collection of data and the interpretation of data, and drafted the manuscript. ML participated in the design and coordination of the study, participated in the interpretation of data and helped draft and revise the manuscript. DH participated in the design of the study, validated the questionnaire, performed the statistical analysis and revised the manuscript. All authors read and approved the final manuscript.

## Supplementary Material

Additional file 1 A Questionnaire of Attitudes Among Pre-Clinical Medical Students, the questionnaire used in this study. Click here for file
