# Supplementary material for: Assessment of the knowledge and attitudes regarding HIV/AIDS among pre-clinical medical students in Israel
Source: BMC Res Notes. 2014 Mar 20;7:168. doi: 10.1186/1756-0500-7-168 (PMC3998113; doi:10.1186/1756-0500-7-168)
Supplement: Additional file 1 — A Questionnaire of Attitudes Among Pre-Clinical Medical Students, the questionnaire used in this study. [file 1756-0500-7-168-S1.pdf]

A Questionnaire of Attitudes Among Pre-Clinical Medical Students

This questionnaire is conducted for study purposes in the course of a final thesis. The questionnaire is anonymous.

Thank you for your cooperation!

1) Demographic Information:

1.1) Gender: 1. Male 2. Female

1.2) Age: _______________

1.3) Country of birth: ______________ 1.4) I immigrated to Israel on: __________

1.5) Sector:

1. Jewish
2. Muslim
3. Christian
4. Druze
5. Beduim
6. Other: _______

1.6) Marital status:

1. Single 2. Married 3. Divorced 4. Other: _______

1.7) Study institute:

1. Technion
2. Tel Aviv University
3. The Hebrew University
4. Be’er Sheva University

1.8) Medical school year:

1. First year 2. Second year 3. Third year

2) Background:

2.1) Do you have any personal acquaintance with a person with HIV/AIDS:

1. Yes 2. No

2.2) The information I have regarding HIV/AIDS comes from the following sources (you may mark more than one answer):

1. Newspapers 2. Magazines 3. Books 4. Medical literature

5. Radio/TV 6. Friends 7. Parents 8. Teachers 9. Doctors

10. Publications on the matter (flyers, ads) 11. Other: _______

2.3) I was exposed to information regarding HIV/AIDS in the course of my studies:

1. Yes 2. No

2.4) I was given this information on year/years:

1. First 2. Second 3. Third

In the course of the following courses: ___________________

2.5) In the course of my studies, I got the chance to meet a person with HIV/AIDS:

1. Yes 2. No

3) Knowledge:

Mark the answer you believe to be most correct:

3.1) In Israel, the most common cause for HIV transmission is (mark only one answer):

1. Prostitutes 2. Intravenous drug users 3. Homosexual relations

4. Heterosexual relations 5. Tattoos and piercings 6. Dental treatments

7. Transmission from mother to fetus

HIV can be transmitted through:		Yes	No	N/A
3.2)	Sexual relations			
3.3)	Infected syringes and needles			
3.4)	Blood transfusion			
3.5)	Mother to child during pregnancy and labor			
3.6)	Mother to child via breastfeeding			
3.7)	Handshake			
3.8)	Mosquito bites			
3.9)	Toilets			
3.10)	Hugging a person with HIV			
3.11)	Saliva of a person with HIV/AIDS			
3.12)	Using the same tableware used by a person with HIV/AIDS			
3.13)	Piercings and tattoos			

Possible treatments:		Yes	No	N/A
3.14)	Nowadays, it is possible to prevent HIV transmission from mother to fetus			
3.15)	The chance of HIV infection after exposure could be lowered if given on time after conducting unprotected sexual intercourse			
3.16)	The chance of HIV infection after exposure could be lowered if given on time after a prick from an infected needle			
3.17)	HIV treatment prolongs the life expectancy of people living with HIV/AIDS			

4) Attitudes:

Mark the answer you find to be most correct according to your stances:

		Highly agree	Agree	Disagree	Highly disagree
4.1)	Only homosexuals could get HIV/AIDS				
4.2)	People with HIV/AIDS got what they deserve				
4.3)	It is hard for me to like people who exposed themselves and society to HIV/AIDS				
4.4)	People with AIDS should be quarantined				
4.5)	Sexual relations should be prohibited for those with HIV/AIDS				
4.6)	Students with HIV/AIDS should be expelled from medical studies				
4.7)	I avoid being among certain people or certain place due to HIV/AIDS				
4.8)	I would like to know more about HIV/AIDS				
4.9)	If I would have had HIV/AIDS, I would be ashamed of it				
4.10)	Other students should be notified if one of the medical students is HIV-positive				
4.11)	I would have a friendship with people with HIV/AIDS				
4.12)	A physician who is HIV-positive should be allowed to work (with the appropriate precautions)				
4.13)	I believe I have the full right to refuse treating a person with HIV/AIDS				
4.14)	I would refuse treating persons with HIV/AIDS				
4.15)	I wish not to treat persons with HIV/AIDS				
4.16)	All patients admitted to the hospital should be tested for HIV				
4.17)	I am concerned that in the future it would be found that HIV infection can be transmitted in ways now thought to be safe				
4.18)	Patients are entitled to know their physicians' HIV status				

	Highly agree	Agree	Disagree	Highly disagree
4.19) All physicians should be HIV tested				
4.20) Health care workers have the right to know their patients' HIV status				
4.21) I would have informed the sexual partner of an HIV positive person about their partners HIV status, even against that patient's request				
4.22) I would warn other medical staff about a patient's HIV status even against that patient's request				
4.23) I would inform an employer about his employee's HIV status, even against the patient's request				
4.24) If given a choice, I would prefer not to treat people with HIV/AIDS				
4.25) Many of the health care workers in Israel are at a high risk of acquiring HIV at work				
4.26) Physicians have the right to refuse treating patients diagnosed with HIV/AIDS				
4.27) There should be routine screening of immigrants for HIV				
4.28) If, as intern, you had to care for a person with HIV/AIDS, would you feel anxious?				
4.29) If as intern, you had to care for a person with HIV/AIDS, would you feel reluctant?				
4.30) I am concerned that working with people who have HIV/AIDS may endanger my health				
4.31) The professional education I received gave me enough information to confidently work with people who have HIV/AIDS				
4.32) The possibility of working with persons with HIV/AIDS will play a role in my choice of specialty				
4.33) The possibility of working with people who have HIV/AIDS will play a role in my choice of place of residence.				
